# Potential clinical drugs as covalent inhibitors of the priming proteases of the spike protein of SARS-CoV-2

**DOI:** 10.1016/j.csbj.2020.08.016

**Published:** 2020-08-26

**Authors:** Qizhang Li, Zhiying Wang, Qiang Zheng, Sen Liu

**Affiliations:** a National “111” Center for Cellular Regulation and Molecular Pharmaceutics, Key Laboratory of Industrial Fermentation (Ministry of Education), Hubei University of Technology, Wuhan 430068, China; b Institute of Biomedical and Pharmaceutical Sciences, Hubei Key Laboratory of Industrial Microbiology, Hubei University of Technology, Wuhan 430068, China

**Keywords:** COVID-19, SCARdock, Drug repurpose, Covalent inhibitors, Drug screening

## Abstract

In less than eight months, the COVID-19 (coronavirus disease 2019) caused by the SARS-CoV-2 (severe acute respiratory syndrome coronavirus 2) virus has resulted in over 20,000,000 confirmed cases and over 700,000 deaths around the world. With the increasing worldwide spreading of this disease, the lack of effective drugs against SARS-CoV-2 infection makes the situation even more dangerous and unpredictable. Although many forces are speeding up to develop prevention and treatment therapeutics, it is unlikely that any de novo drugs will be available in months. Drug repurposing holds the promise to significantly save the time for drug development, since it could use existing clinic drugs to treat new diseases. Based on the “steric-clashes alleviating receptor (SCAR)” strategy developed in our lab recently, we screened the library of clinic and investigational drugs, and identified nine drugs that might be repurposed as covalent inhibitors of the priming proteases (cathepsin B, cathepsin L, and TMPRSS2) of the spike protein of SARS-CoV-2. Among these hits, five are known covalent inhibitors, and one is an anti-virus drug. Therefore, we hope our work would provide rational and timely help for developing anti-SARS-CoV-2 drugs.

## Graphical abstract

**Figure:**
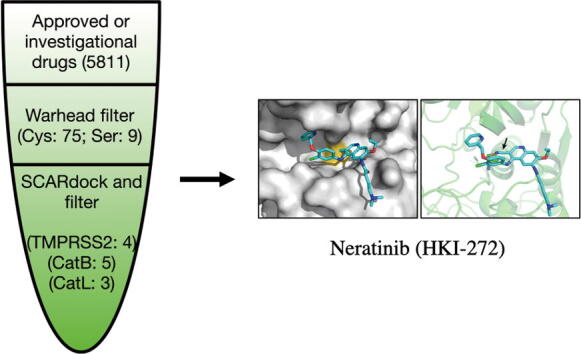

## 1 Introduction

Since its outbreak in December 2019, the COVID-19 (coronavirus disease 2019) disease from the infection of the SARS-CoV-2 (severe acute respiratory syndrome coronavirus 2) virus has caused over 20,000,000 confirmed cases and over 700,000 deaths in over 180 countries/regions as of August 12, 2020 (https://coronavirus.jhu.edu). Although most COVID-19 patients can recover from the disease, some of the severe patients might suffer from long-term health issues including irreversible lung damages [1] and fertility compromise [2]. In addition to the devastating health crisis, the ongoing COVID-19 pandemic is also inflicting heavy losses on the global economy due to city lockdowns and glitches in supply chains (https://www.imf.org/en/Topics/imf-and-covid19). Facing the escalating risk from COVID-19, the whole world is intensively working on the discovery of prevention and treatment options for SARS-CoV-2 infection [3]. According to the statistics of the Milken Institute (https://milkeninstitute.org/covid-19-tracker), over 200 vaccines and 300 treatment options for COVID-19 are under development worldwide as of August 12, 2020. Nonetheless, the reality is that neither de novo drug discovery nor vaccine development is a task that can be accomplished in several months or several years even if the huge monetary cost is guaranteed [4]. Therefore, to save the world from the current SARS-CoV-2 crisis, a more promising option would be looking for known drugs that could be used to treat COVID-19 patients.

To develop new uses for a drug beyond its original use, or drug repurposing (also known as drug repositioning, reprofiling, redirecting, or rediscovering), can significantly cut the time and money costs in drug development [5]. For example, drug repurposing has been successfully applied on the use of sildenafil in treating erectile dysfunction and the anti-cancer uses of thalidomide [6]. Since the outbreak of COVID-19, a number of clinic drugs have been repurposed for the treatment of the infected patients, such as lopinavir/ritonavir, chloroquine, ribavirin, and arbidol [7]. However, none of these tested drugs had confirmed efficacy yet. Among them, remdesivir, an investigational drug developed to treat the Middle East respiratory syndrome coronavirus (MERS-CoV), is one of the most promising. The latest clinical study showed that remdesivir was superior to placebo in shortening the time to recovery in adults hospitalized with Covid-19 [8], but efficacious drugs for treating COVID-19 patients are still urgently needed for the current SARS-CoV-2 pandemic.

The spike (S) protein of coronaviruses facilitates viral entry into cells by binding to receptors and driving the fusion of cell membranes [9]. Prior to be functional, the S protein needs to be cleaved and activated by the cellular proteases of the host cell [9]. According to a recent study [9], both the endosomal cysteine proteases cathepsin B/L (CatB/L) and the transmembrane protease serine type 2 (TMPRSS2) can prime the S protein of SARS-CoV-2. Meanwhile, TMPRSS2 can cleave carboxypeptidase angiotensin-converting enzyme 2 (ACE2), the host cellular receptor of the S protein, to augment viral infectivity [10]. Therefore, similar with SARS-CoV [11] and MERS-CoV [12], the simultaneous inhibition of CatB/L and TMPRSS2 might be necessary for completely blocking the cellular entry of SARS-CoV-2 [9].

Historically, the drug discovery practice mainly focuses on non-covalent drugs due to potential off-target effects and toxicity issues of irreversible covalent drugs [13]. However, recent years have witnessed the resurgence of covalent drugs because many people have realized that compared to non-covalent drugs, covalent drugs might have extra advantages including: (i) better biochemical efficiency since they are more competitive than non-covalent endogenous substrates and co-factors [14]; (ii) lower patient burden and less drug resistance due to lower and less frequent dosing [14]; (iii) improved target specificity upon careful structural designs targeting specific residues [15], [16]. To help the discovery of covalent drugs, we previously established a “steric-clashes alleviating receptor (SCAR)” strategy [17] for the *in silico* docking and screening of covalent drugs enlightened by *in silico* protein design [18]. More recently, we demonstrated that our SCARdock method is also useful in drug repurposing [19], [20].

In this study, we set out to use SCARdock to identify possible covalent inhibitors of CatB, CatL, and TMPRSS2 from clinic and investigational drugs. We filtered a ZINC database (http://zinc15.dock.org) containing approved and in-trial drugs with known warhead groups targeting cysteine (CatB/CatL) or serine (TMPRSS2). Then, SCARdock was used to computationally screen potential covalent inhibitors of human CatB, CatL, and TMPRSS2. After careful filtering and evaluation, we identified five (trapoxin B, neratinib, HKI-357, domatinostat and (*Z*)-dacomitinib) potential covalent inhibitors for CatB, three (neratinib, HKI-357 and (*Z*)-dacomitinib) for CatL, and four (lodoxamide, aceneuramic acid, (*S*)-boceprevir and (*R*)-boceprevir) for TMPRSS2. Interestingly, neratinib, HKI-357 and (*Z*)-dacomitinib could be covalent inhibitors for both CatB and CatL. More importantly, trapoxin B [21], neratinib [22], HKI-357 [22], (*Z*)-dacomitinib [23] and boceprevir [24] are known to function as covalent inhibitors. Therefore, our work might provide prominent help for discovering anti-virus drugs to combat the current COVID-19 threat.

## 2 Materials and methods

### 2.1 Preparation of the screening library

The structure files of the screening compounds were downloaded as mol2 files from the ZINC15 database (http://zinc15.docking.org). The 3D conformations were protonated at physiological pH, and biologically relevant tautomers were generated for each molecule as described in ZINC15 [25]. The “in-trials” catalog (2019-04-22 version) was downloaded, which contained 5811 approved or investigational (clinically tested but not approved) drugs worldwide. MGLTools (version 1.5.6) was used to generate the PDBQT files from the mol2 files for docking.

### 2.2 Preparation of protein structures

The 3D structures of the indicated proteins were downloaded from the RCSB database (http://www.rcsb.org/). The homology model of TMPRSS2 was obtained from the SWISS-MODEL repository (https://swissmodel.expasy.org/repository/uniprot/O15393), which was built from the homologous protein TMPRSS1 (PDB ID: 5CE1). As mentioned previously [17], the protein structures were relaxed in Rosetta 3 [26] to eliminate possible structural conflicts. MGLTools (version 1.5.6) was used to generate the corresponding PDBQT files for docking.

### 2.3 Preparation of existing inhibitors

The small molecule inhibitors were extracted from the complex structures mentioned above. The structures were visually checked, and incorrect bonds/atoms were manually corrected in IQmol (version 2.14.0). MGLTools (version 1.5.6) was used to generate the corresponding PDBQT files for docking.

### 2.4 SCARdock screening and filtering

To prepare the SCAR proteins [17] used in SCARdock, Cys29 of CatB, Cys25 of CatL, and Ser441 of TMPRSS2 were computationally mutated to Gly to eliminate the sidechain clashes. The small molecules were docked into the corresponding pockets of the proteins with AutoDock Vina (version 1.1.2) [27]. The docking process did not consider the flexibility of the protein. The space coordinates of the S atom (for Cys) or the O atom (for Ser) in the wild-type protein were used for calculating the atom distances of the bonding atoms in the warhead groups. The distance cutoff between the bonding atoms and the S/O atom in the protein was set to 1.8 Å, indicating that the conformation with a distance above 1.8 Å is not accepted. Since the results (Table 1) had distances between 1.2 and 1.8 Å, no score punishment for steric conflict was applied for the cases in this study. For each ligand, top 10 poses were used for evaluation. The score cutoff was set as −7.5 for CatB, −7.0 for CatL, and −6.0 for TMPRSS2.

**Table 1:** The drugs repurposed as potential covalent inhibitors of the indicated target proteins using SCARdock.

Target Proteins	ZINC ID	Atom distance^a^ (Å)	Docking Score^b^	Pose rank^b^	Warhead	CAS number	DrugBank ID	Drug name	Approved or Investigational treatment	SCAR enriching score^c^
CatB	ZINC000003925368	1.2	−7.7	7	epoxide	133155-90-5	–	Trapoxin B	–	−0.5
	ZINC000003916214	1.6	−7.9	2	nitrile	698387-09-6	DB11828	Neratinib (HKI-272)	Breast cancer	0.3
	ZINC000028124370	1.6	−8.2	1	nitrile	848133-17-5	DB13002	HKI-357	Investigational	0.4
	ZINC000034851244	1.6	−8.5	6	amide	910462-43-0	DB13101	Domatinostat (4SC-202)	Advanced hematologic malignancies	0
	ZINC000095566645	1.8	−8.2	3	amide	–	–	(*Z*)-Dacomitinib	–	0
CatL	ZINC000003916214	1.7	−7.1	5	nitrile	698387-09-6	DB11828	Neratinib (HKI-272)	Breast cancer	−0.1
	ZINC000028124370	1.6	−7.7	3	nitrile	848133-17-5	DB13002	HKI-357	Investigational	0.4
	ZINC000095566645	1.8	−7.5	5	amide	–	–	(*Z*)-Dacomitinib	–	−0.1
TMPRSS2	ZINC000002000707	1.6	−6.8	1	α-ketoacid	63610-09-3	DB06794	Lodoxamide	Ocular hypersensitivity reactions	0.3
	ZINC000004214715	1.8	−6.4	1	α-ketoacid	131-48-6	DB11797	Aceneuramic Acid	Hereditary inclusion body myopathy	0
	ZINC000014210455	1.5	−6.8	1	α-ketoamide	394730-60-0	DB08873 DB05665	(*S*)-Boceprevir	Antiviral medication, chronic hepatitis C	0.1
	ZINC000014210457	1.8	−6.4	4	α-ketoamide	394730-60-0	DB08873 DB05665	(*R*)-Boceprevir	Antiviral medication, chronic hepatitis C	−1.3

a The distance between the bonding atom of the drug and the bonding atom of the residue in the protein.

b Docking scores and pose ranks are listed for the reported poses.

c Calculated as the top docking score of the drug docked to the wild-type protein minus the listed docking score.

## 3 Results

### 3.1 The overall screening results

**Fig. 1:**
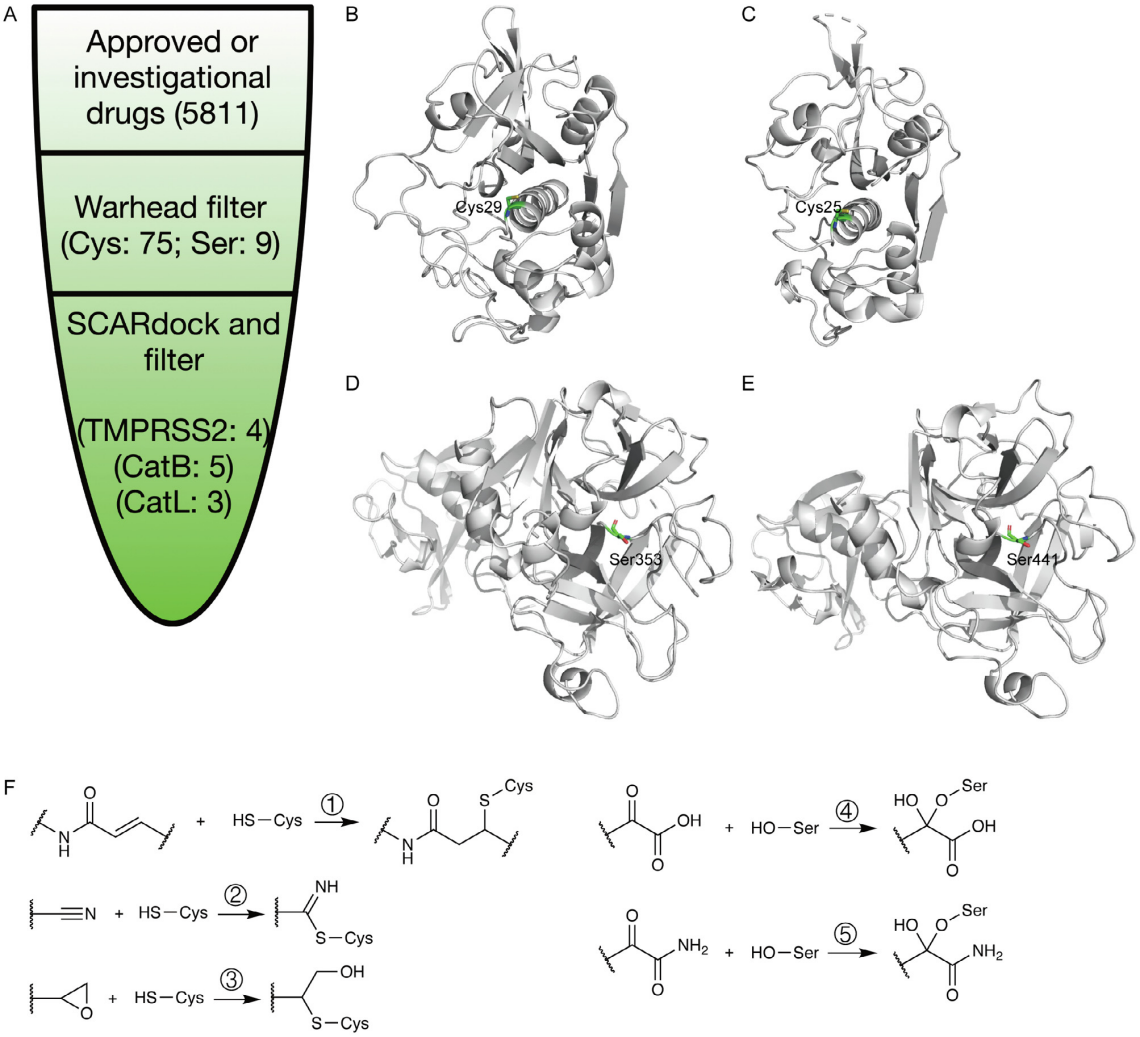
(A) A schematic diagram of the screening workflow in this study. (B-E) Structures of cathepsin B (B), cathepsin L (C), TMPRSS1 (D) and TMPRSS2 (E). Proteins are shown in gray ribbons. The nucleophilic cysteines are shown in sticks and colored in green. (F) Reaction mechanisms of the amide (1), nitrile (2), epoxide (3), α-ketoacid (4) and α-ketoamide (5) warheads with target residues. (For interpretation of the references to colour in this figure legend, the reader is referred to the web version of this article.)

Among the 5811 approved or investigational clinic drugs, we obtained 75 containing potential reactive groups (warheads) targeting cysteine and 9 containing potential warheads targeting serine (Fig. 1A). Based on previous studies [28], [29], Cys29 of CatB and Cys25 of CatL are nucleophilic and can covalently bind to electrophilic ligands (Fig. 1B & 1C). Following the SCARdock protocol [17], these reactive residues were computationally mutated to glycine to generate the SCAR proteins for docking. As of TMPRSS2, the homology model showed that its Ser441 is comparable to the residue Ser353 of TMPRSS1 (Hepsin) (Fig. 1D & 1E). Since previous studies showed that TMPRSS1-Ser353 covalently bound to substrates [30], TMPRSS2-Ser441 was mutated to glycine for SCARdock.

From the RCSB database, we obtained four X-ray structures of human CatB and seventeen X-ray structures of human CatL (Supplementary Table 1). To identify the most suitable structures for SCARdock, we docked all the known inhibitors in these complex structures to each SCAR proteins of the respective PDB structure. After evaluating the recaptured X-ray poses of the docked inhibitors (Supplementary Table 1), the protein structure from 1CSB (PDB ID) was chosen for CatB, and the protein structure from 5MAE (PDB ID) was chosen for CatL. The human TMPRSS2 structure was obtained from the SWISS-MODEL repository since there were no X-ray structures available. These structures were then used for SCARdock screening, and after distance and score filtering, we identified five potential covalent inhibitors for CatB, three for CatL, and four for TMPRSS2 (Table 1). Overall, three cysteine covalent warheads and two serine covalent warheads were observed in the identified drugs (Fig. 1F).

### 3.2 Potential covalent inhibitors targeting CatB

**Fig. 2:**
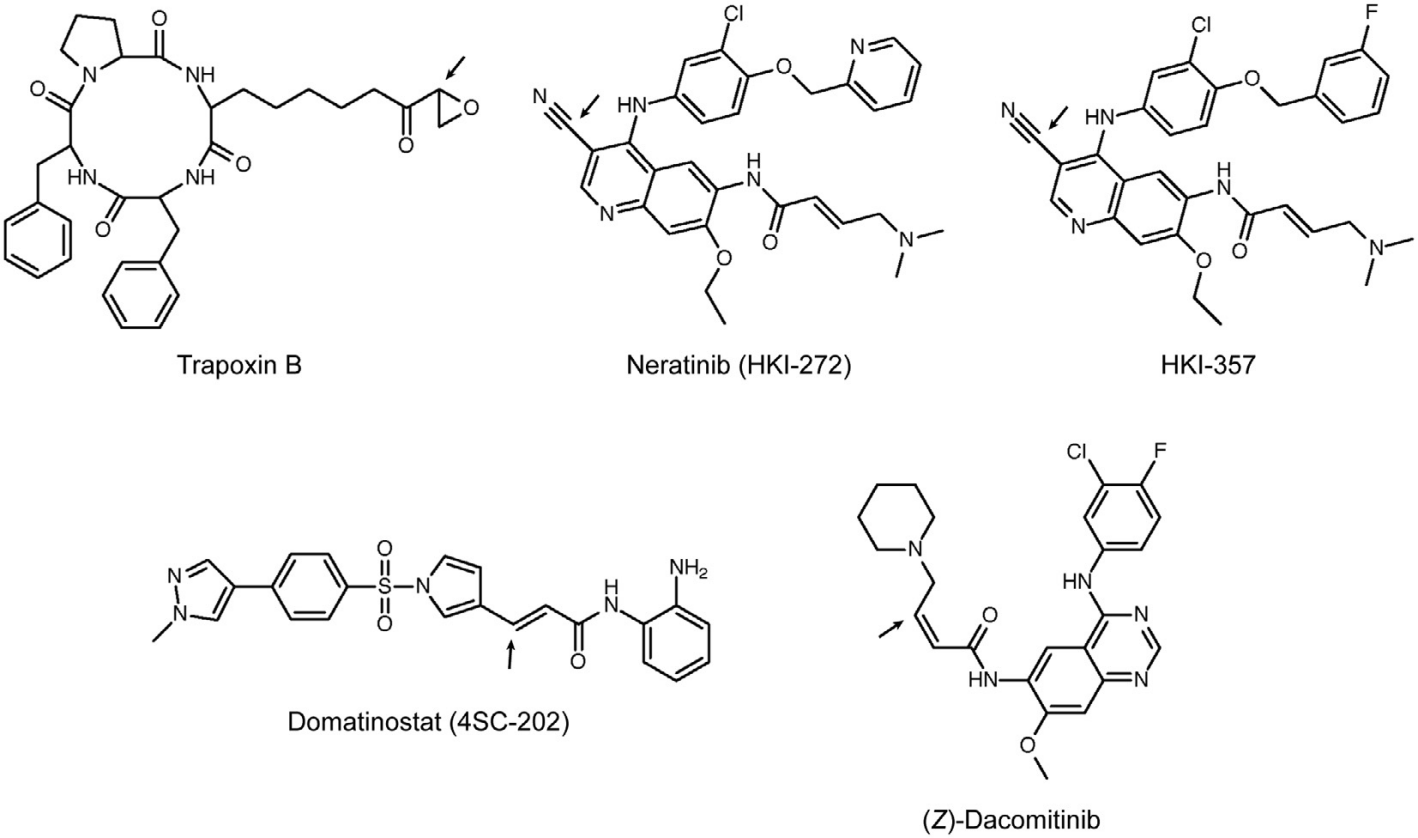
Structures of the SCARdock hits for CatB. The bonding atoms in the warheads are pointed out by arrows.

**Fig. 3:**
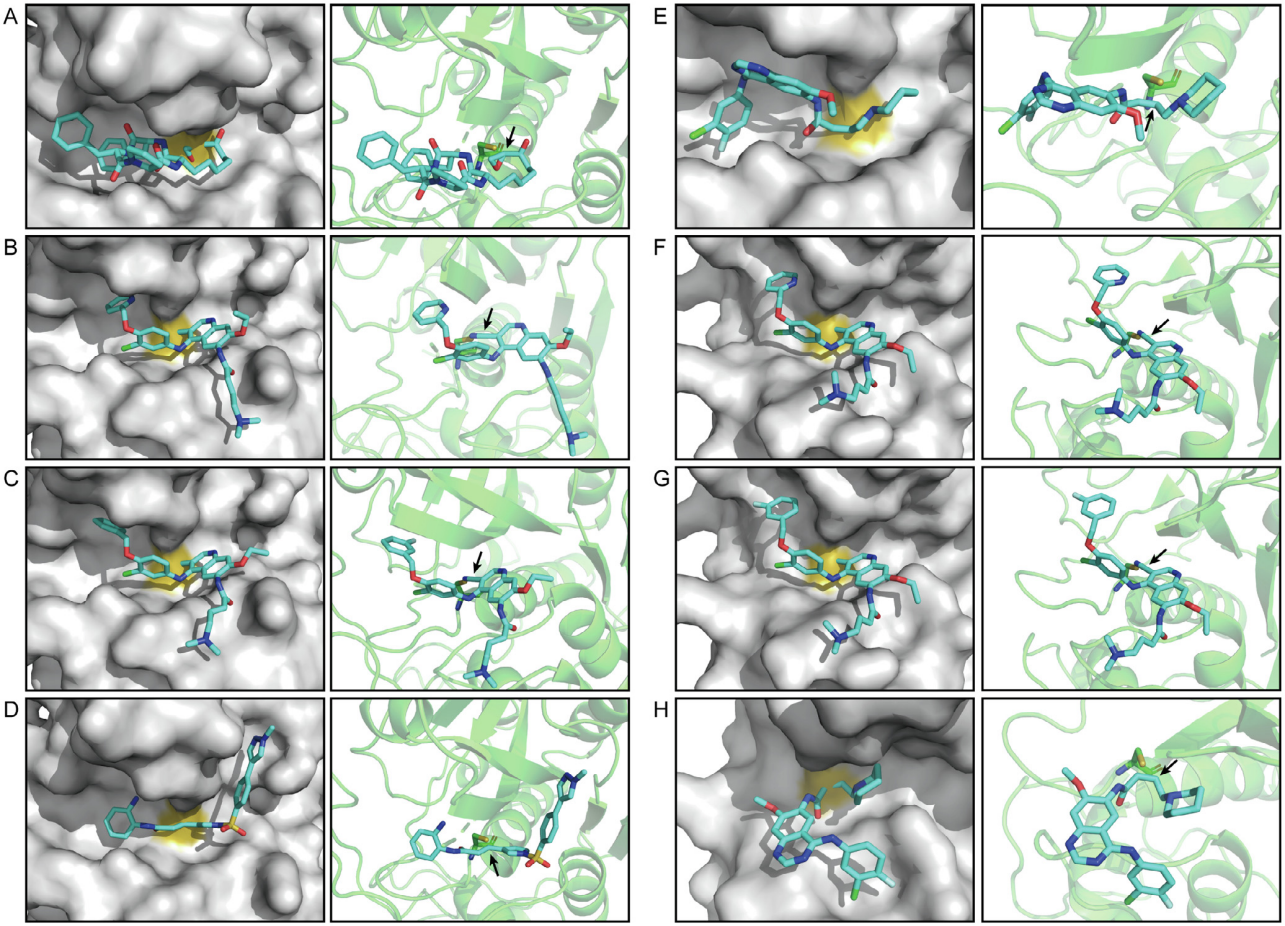
Docked poses of the identified hits of CatB (A-E) and CatL (F-H). The proteins are shown in surface or ribbons, and the drugs are shown in sticks. The cysteine residues for covalent binding are colored in yellow. The displayed items for CatB are trapoxin B (A), neratinib (HKI-272) (B), HKI-357 (C), domatinostat (4SC-202) (D) and (*Z*)-dacomitinib (E). The displayed items for CatL are neratinib (HKI-272) (F), HKI-357 (G) and (*Z*)-dacomitinib (H). The putative covalent bonding atoms in the drugs are indicated by arrows. (For interpretation of the references to colour in this figure legend, the reader is referred to the web version of this article.)

**Fig. 4:**
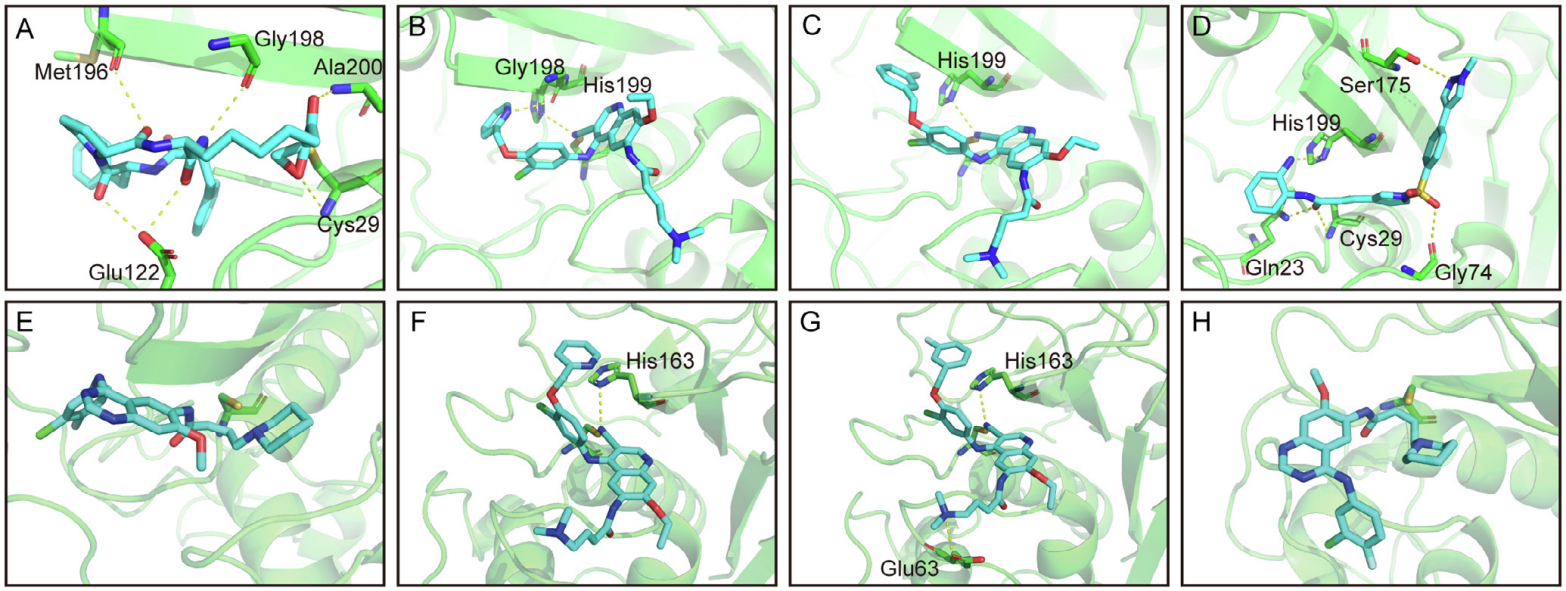
The binding details between the SCARdock hits and CatB/CatL. The docked poses are as same as in Fig. 3. For CatB: trapoxin B (A), neratinib (HKI-272) (B), HKI-357 (C), domatinostat (4SC-202) (D) and (*Z*)-dacomitinib (E); for CatL: neratinib (HKI-272) (F), HKI-357 (G) and (*Z*)-dacomitinib (H). The proteins are shown in ribbons with key residues shown in sticks, and the drugs are shown in sticks. Dashed lines represent hydrogen bonds.

The five potential covalent drugs identified for CatB are trapoxin B, neratinib (HKI-272), HKI-357, domatinostat (4SC-202) and (*Z*)-dacomitinib (Fig. 2 & Table 1). Trapoxin B contains an epoxide warhead. For this warhead, the nucleophilic attack might occur on the ring carbon next to the carbonyl carbon, and then a covalent bond can form between the sulfur atom of Cys29 and the bonding carbon of the oxirane moiety, accompanied by the ring opening and the formation of a hydroxyl group (Fig. 1F & Fig. 3A). Neratinib (HKI-272) and HKI-357 contain a nitrile warhead. For this warhead, a covalent thioimidate bond might form at the electrophilic nitrile carbon after the attack of the cysteine sulfur atom (Fig. 3B & 3C). Domatinostat (4SC-202) and (*Z*)-dacomitinib are amide-based ligands. A covalent bond might form between the β-carbon and the cysteine sulfur atom (Fig. 3D & 3E). In addition to these possible covalent bonds, the non-covalent scaffolds of these drug can also form suitable hydrogen bonds with CatB except (*Z*)-dacomitinib (Fig. 4A-4F).

### 3.3 Potential covalent inhibitors targeting CatL

Three drugs, i.e. neratinib (HKI-272), HKI-357 and (*Z*)-dacomitinib, were identified as potential CatL covalent inhibitors (Table 1). Interestingly, these drugs were also predicted as CatB covalent inhibitors (Table 1). This result is not unexpected though, since CatB and CatL are homologous and have very similar 3D structures (Fig. 1B & 1C). Thus, neratinib (HKI-272), HKI-357 and (*Z*)-dacomitinib are potential covalent inhibitors for both CatB and CatL. Although the docked poses were slightly different on these two proteins, the warheads of these drugs were also at the positions suitable for covalent bonding (Fig. 3F-H). Additionally, possible hydrogen bonds can also form between these ligands and CatL except (*Z*)-dacomitinib (Fig. 4F-4H).

### 3.4 Potential covalent inhibitors targeting TMPRSS2

**Fig. 5:**
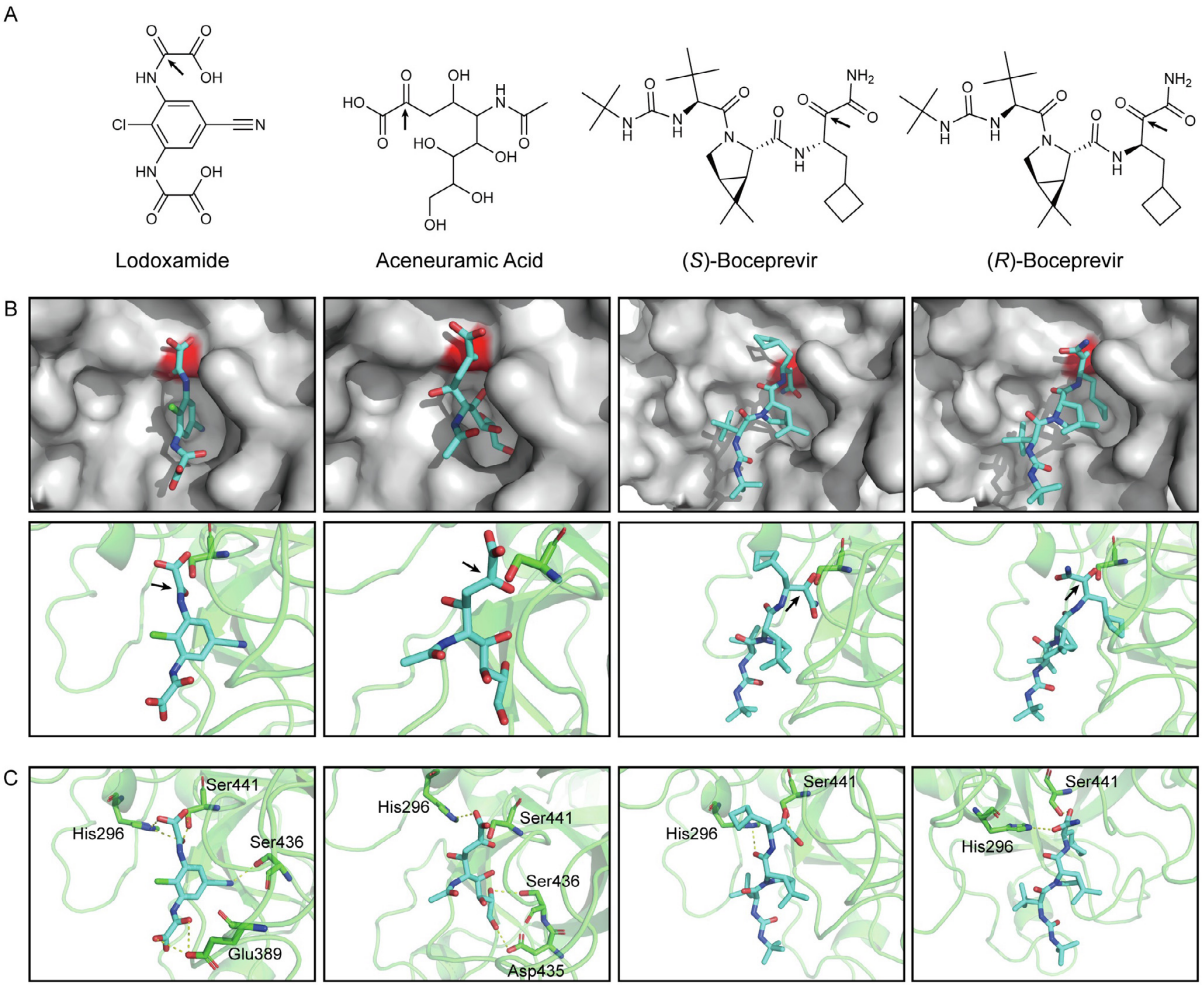
The identified hits for TMPRSS2. (A) Structures of lodoxamide, aceneuramic acid, (*S*)-boceprevir and (*R*)-boceprevir. (B) The docked poses of the hits in TMPRSS2. The proteins are shown in surface or ribbons, and the drugs are shown in sticks. The serine residues for covalent binding are colored in red. The putative covalent bonding atoms in the drugs are indicated by arrows. (C) The binding details between the SCARdock hits and TMPRSS2. The proteins are shown in ribbons with key residues shown in sticks, and the drugs are shown in sticks. Dashed lines represent hydrogen bonds. (For interpretation of the references to colour in this figure legend, the reader is referred to the web version of this article.)

Four compounds, i.e. lodoxamide, aceneuramic acid, (*S*)-boceprevir and (*R*)-boceprevir, were identified as potential TMPRSS2 covalent inhibitors (Fig. 5A). Lodoxamide, aceneuramic acid and aleplasinin possess an α-ketoacid group, whereas (*S*)- and (*R*)-boceprevir have an α-ketoamide group. Both of these groups could be used as covalent warheads targeting serine (Fig. 1F). As shown in Fig. 5B, the warheads in these inhibitors are positioned well for the covalent bonding between the bonding atoms and the hydroxyl group of TMPRSS-Ser441. In addition, multiple hydrogen bonds were also observed between the inhibitors and the protein (Fig. 5C).

## 4 Discussion and conclusions

In his TED talk five years ago, Bill Gates warned that “if anything kills over 10 million people in the next few decades, it’s most likely to be a highly infectious virus rather than a war.” The ongoing COVID-19 pandemic caused by the SARS-CoV-2 virus is undoubtedly reminding us his warning was not a hoax. The high infection rate and mortality ratio of COVID-19 are unexpected [31], and the SARS-CoV-2 virus has made 2020 a difficult year for a lot of people in the world. Although this virus would unlikely kill over 10 million people, it is still posing an unprecedent threat to both the health and the economy of the whole world due to the shortage of effective prevention and treatment therapeutics so far. In the regular research pipeline, the development of a new drug will cost over 10 years, but drug repurposing might significantly save the time [5], which might be the best chance for combating the current COVID-19 pandemic in time [32].

During the infection of coronaviruses, the spike (S) protein mediates host recognition and binding. However, the S protein needs to be cleaved and primed by the host cell before the virus can enter and hijack the host cell [9]. CatB, CatL, and TMPRSS2 of the human cell can prime the S protein of SARS-CoV-2 [9]. Therefore, as the other similar proteases [33], targeting these priming proteases might be an effective choice to disrupt the infection of this virus. Based on our recent work [17], [19], [20], we adopted the SCARdock protocol to repurpose clinic drugs as potential inhibitors of these priming proteases in this study. We identified several clinic drugs that might be useful as the covalent inhibitors of CatB, CatL, and TMPRSS2.

Neratinib (HKI-272), HKI-357 and dacomitinib are identified as potential covalent inhibitors for both CatB and CatL. All of these three drugs have an acrylamide group that is suitable for binding cysteine covalently, but neratinib (HKI-272) and HKI-357 also have an additional nitrile group, another potential covalent warhead targeting cysteine (Fig. 1F). More interestingly, all these drugs are covalent pan-HER (human epidermal growth factor receptor) kinase inhibitors targeting the nucleophilic cysteine in the ATP binding site of EGFR and/or HER2 [34]. This fact indicated that these three molecules are electrophilic, and it is highly possible that they can form covalent bonds with the indicated cysteine residues of CatB and CatL if the non-covalent binding affinity is high enough. Nontheless, we want to note that in our docking results, the nitrile groups, instead of the acrylamide groups, of neratinib (HKI-272) and HKI-357 were close to the cysteine residues of CatB/CatL in the top 10 poses (Fig. 3B, 3C, 3F & 3G). However, there were docked poses of these two drugs with suitably positioned acrylamide groups in less optimal poses (Supplementary Fig. 1). In addition, domatinostat (4SC-202) and trapoxin B were identified as potential CatB covalent inhibitors. Interestingly, both domatinostat (4SC-202) and trapoxins B are the inhibitors of histone deacetylases (HDACs), and they have been used for the treatment of advanced hematological malignancies [35], [36]. Therefore, neratinib (HKI-272), HKI-357 and dacomitinib might be the most attractive drugs worth experimental validation for CatB/CatL among all of these hits.

As of the identified hits of TMPRSS2, the most attractive drug might be boceprevir, which is a first-generation inhibitor of hepatitis C virus non-structural protease 3 (HCV NS3) [37]. Based on the docking score and the SCAR enriching score (Table 1), (*S*)-boceprevir might be better than (*R*)-boceprevir. Since boceprevir has anti-virus activity, it will be worth a try for SARS-CoV-2 infection. In addition, lodoxamide is a mast cell stabilizing compound with anti-inflammatory activity [38], aceneuramic acid is used for the treatment of hereditary inclusion body myopathy [39], and aleplasinin was developed to treat Alzheimer disease [40]. A note is that the screening of TMPRSS2 inhibitors was based on a homology model of human TMPRSS2, which might make the result not as reliable as the CatB/L cases. However, it would be worthwhile to test if these three drugs will have anti-SARS-CoV-2 activities.

**Fig. 6:**
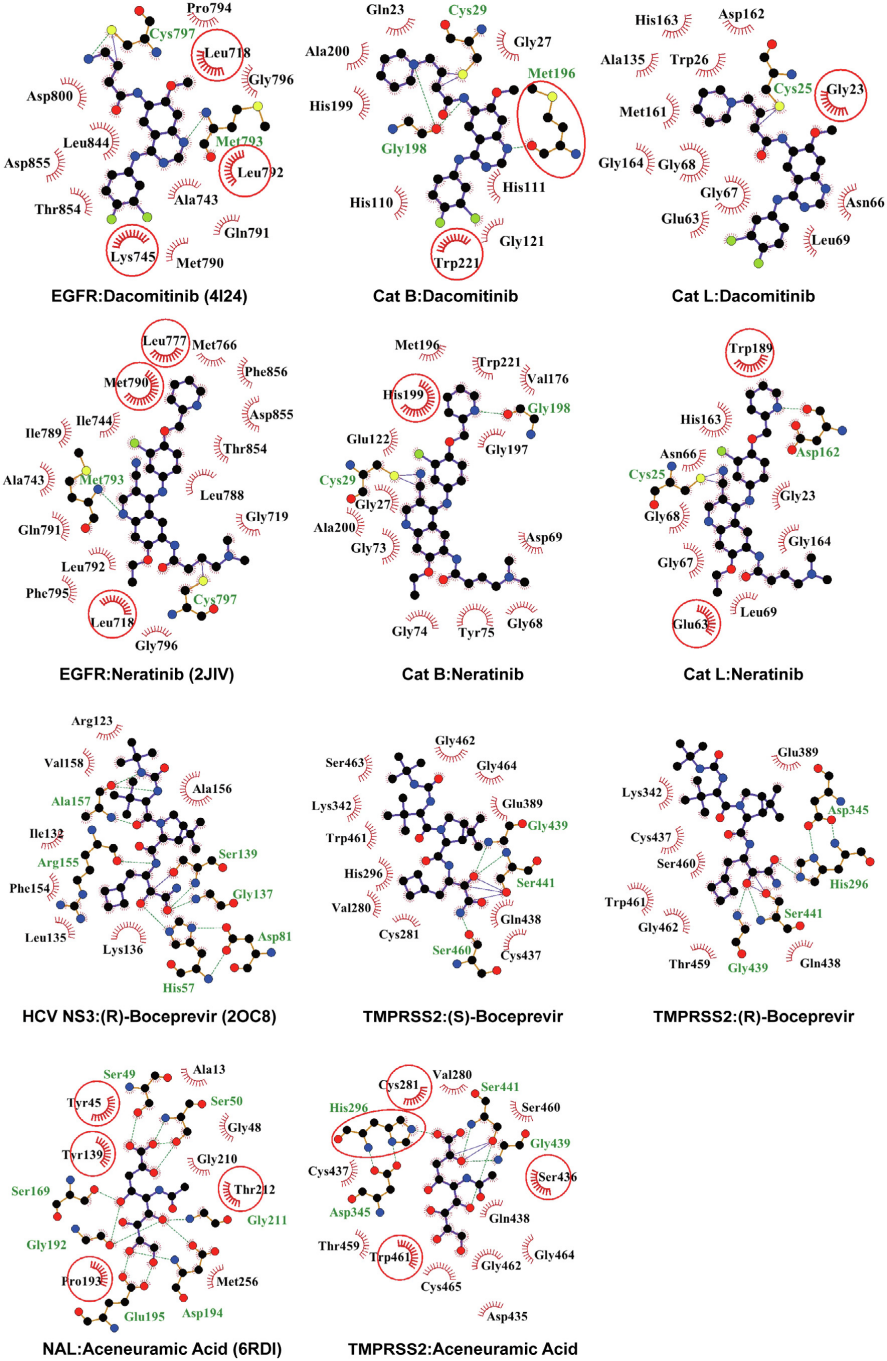
The comparison of the interactions of the identified hits with their targets. The plots were prepared with LigPlot+. Ligands and protein side chains are shown in ball-and-stick representation, with the ligand bonds colored in purple. Hydrogen bonds are shown as green dotted lines, while the spoked arcs represent protein residues making nonbonded contacts with the ligands. The red circles and ellipses indicate protein residues that are in equivalent 3D positions when the structural models are superposed. The PDB codes of the experimental complex structures were shown in the parentheses. Part of the structure of dacomitinib was missing in the complex structure with EGFR (PDB ID: 4I24). (For interpretation of the references to colour in this figure legend, the reader is referred to the web version of this article.)

A theoretical method to evaluate if a ligand could bind to two different proteins is to compare the similarity of the binding pockets of the target proteins [41]. Presumably, the protein–ligand interactions between a same ligand and different proteins should be similar, although the extent to which should vary on a case-by-case basis [41]. Therefore, to validate the binding potential of our computational hits with the target proteins, the ligand–protein interactions were generated (Fig. 6). For meaningful comparison, only the hits with experimental complex structures available were included. As shown in Fig. 6, compared to experimental complex structures, the docked ligands had similar hydrophobic interactions and hydrogen bonds with the target proteins. This analysis also showed that (*S*)-boceprevir formed more interactions with TMPRSS2 than (*R*)-boceprevir did, which, in agreement with their docking scores and SCAR enriching scores (Table 1), supported the conclusion that (*S*)-boceprevir might be better than (*R*)-boceprevir for inhibiting TMPRSS2.

Taken together, using our SCARdock protocol, we identified nine drugs that might be repurposed as the covalent inhibitors of the priming proteases of the S protein of SARS-CoV-2. Among these nine drugs, neratinib (HKI-272), HKI-357, dacomitinib, and boceprevir might be highly potential with moderate side effects (Supplementary Table 2). We hope our work will provide the scientific community additional options for tackling the unprecedented COVID-19 threat.

## Author contributions

S.L. conceived the idea and did the computational work. Z.Y.W., S.L., and Q.Z. did the computational work. S.L., Q.Z.L., and Z.Y.W. analyzed and interpreted the data. Q.Z.L., Z.Y.W., and S.L. wrote the manuscript. All authors reviewed and approved the submitted manuscript.

## Funding

This work was supported by the grants from National Natural Science Foundation of China (31670768, 31971150), Hubei Provincial Science and Technology Department (2019CFA069), Wuhan Science and Technology Bureau of China (2018060401011319), and Hubei University of Technology.

## CRediT authorship contribution statement

**Qizhang Li:** Formal analysis, Data curation, Writing - original draft. **Zhiying Wang:** Methodology, Validation, Formal analysis, Visualization. **Qiang Zheng:** Methodology. **Sen Liu:** Conceptualization, Methodology, Resources, Supervision, Project administration, Funding acquisition.

## Declaration of Competing Interest

The authors declare that they have no known competing financial interests or personal relationships that could have appeared to influence the work reported in this paper.
