# Supplementary material for: Potential clinical drugs as covalent inhibitors of the priming proteases of the spike protein of SARS-CoV-2
Source: Comput Struct Biotechnol J. 2020 Aug 26;18:2200–8. doi: 10.1016/j.csbj.2020.08.016 (PMC7448953; doi:10.1016/j.csbj.2020.08.016)
Supplement: Supplementary data 1 [file mmc1.pdf]

Potential clinical drugs as covalent inhibitors of the priming proteases of the spike protein of SARS-CoV-2

Qizhang Li[1,2,$], Zhiying Wang[1,2,$], Qiang Zheng[1,2], Sen Liu[1,2,*]

[1]National "111" Center for Cellular Regulation and Molecular Pharmaceutics, Key Laboratory of Industrial Fermentation (Ministry of Education), Hubei University of Technology, Wuhan 430068, China

[2]Institute of Biomedical and Pharmaceutical Sciences, Hubei Key Laboratory of Industrial Microbiology, Hubei University of Technology, Wuhan, 430068, China

[$] These authors had equal contributions.

*** Correspondence:**

Sen Liu

senliu.ctgu@gmail.com

Supplementary Table 1. PDB IDs and the structural information of human cathepsin B/L in complex with different ligands. Docking scores, number of recaptured poses, and pose rank are from SCARdocking. For each protein, the covalent ligands from those PDB structures were extracted and docked to each PDB structure.

PDB ID	Protein	Resolution (Å)	Organism	Covalent Residues	Number of Recaptured Inhibitors[a]	Docking Score[b]	Pose Rank[b]	Atom Distance[c] (Å)
1CSB	Cathepsin B (CatB)	2.0	Homo sapiens	Cys29	2	-8.5	2	1.8
1GMY		1.9	Homo sapiens	Cys29	1	-7.7	7	1.8
2IPP		2.15	Homo sapiens	Cys29	1	-	-	1.8
6AY2		1.6	Homo sapiens	Cys29	1	-	-	1.7
2XU1	Cathepsin L (CatL)	1.45	Homo sapiens	Cys25	5	-8.3	1	1.9
2XU3		0.9	Homo sapiens	Cys25	5	-8.2	1	1.8
2XU4		1.12	Homo sapiens	Cys25	8	-7.8	1	1.8
2XU5		1.6	Homo sapiens	Cys25	7	-8.1	1	1.8
2YJ2		1.15	Homo sapiens	Cys25	5	-8.4	1	1.8
2YJ8		1.3	Homo sapiens	Cys25	6	-8.3	1	1.8
2YJ9		1.35	Homo sapiens	Cys25	5	-8.8	1	1.8
2YJB		1.4	Homo sapiens	Cys25	6	-8.8	1	1.8
2YJC		1.14	Homo sapiens	Cys25	5	-8.3	1	1.8
3HHA		1.27	Homo sapiens	Cys25	4	-7.1	2	1.9
3HWN		2.33	Homo sapiens	Cys25	0	-	-	1.7
3OF8		2.2	Homo sapiens	Cys25[d]	1	-	-	1.8
3OF9		1.76	Homo sapiens	Cys25[d]	2	-	-	1.8
5F02		1.43	Homo sapiens	Cys25	5	-7.9	1	1.8
5MAE		1.0	Homo sapiens	Cys25	11	-6.3	4	1.7
5MAJ		1.0	Homo sapiens	Cys25	8	-8.1	2	1.8
5MQY		1.13	Homo sapiens	Cys25	6	-	-	1.8

-: No docked poses close to X-ray conformations.[a] Number of recaptured known inhibitors in cross SCARdock.[b] Docking scores and pose ranks are listed for the identified poses. These data are from the SCARdocking results on the selected PDB structures (1CSB for CatB, and 5MAE for CatL). [c] The distance between the bonding atom in the drug and the bonding atom of the protein residue. [d] Corrected residue numbers for these two structures. The residue numbers are 26 in the PDB structures.

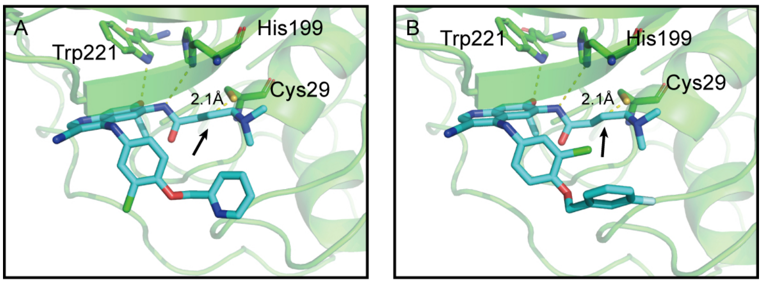

CatB-HKI-272
docking score:-7.6
pose rank:4

CatB-HKI-357
docking score:-7.6
pose rank:5

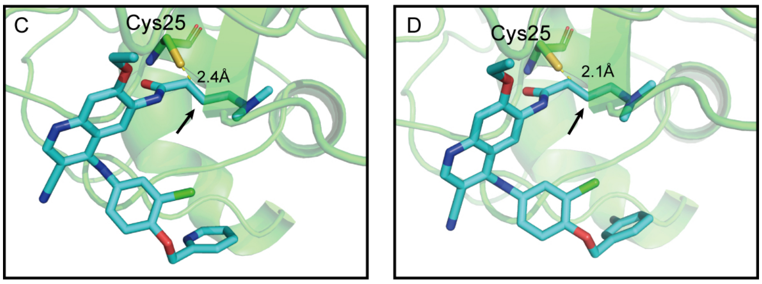

CatL-HKI-272
docking score:-7.6
pose rank:4

CatL-HKI-357
dockin score:-7.1
pose rank:5

Supplementary Figure 1: Alternative docking poses of Neratinib (HKI-272) and HKI-357 on CatB and CatL with the acrylamide warhead close to the reactive cysteines. The drugs were docked with SCARdocking, and the wild-type proteins without mutations are shown for distance comparison.

Supplementary Table 2. The information of the drugs repurposed as potential covalent inhibitors.

Drug Name	ZINC ID	CAS number	DrugBank ID	NCT Number	Dose	Administration	half-life	Adverse Events
Trapoxin B	ZINC000003925368	133155-90-5	-	-	-	-	-	-
Neratinib (HKI-272)	ZINC000003916214	698387-09-6	DB11828	NCT00397046 NCT00958724 NCT01128842 NCT01827267 NCT01142063 NCT01808573 NCT00878709	240 mg once daily	Orally	13.9 h	diarrhea, hepatotoxicity, vomiting, dehydration, cellulitis, renal failure, erysipelas, alanine aminotransferase increase, aspartate aminotransferase increase, nausea, fatigue, and abdominal pain
HKI-357	ZINC000028124370	848133-17-5	DB13002	NCT00550381	-	Orally	-	-
Domatinostat (4SC-202)	ZINC000034851244	910462-43-0	DB13101	NCT01344707	200 mg twice daily	Orally	146 h	hematological laboratory decrease, liver enzyme increase, pulmonary embolism, hypercalcemia, rash, diarrhea, and nausea
(*Z*)-Dacomitinib	ZINC000095566645	-	-	NCT00783328	45 mg once daily	Orally	80.0 h	rash, diarrhea, and nausea
Lodoxamide	ZINC000002000707	63610-09-3	DB06794	-	0.1%	Eye drops	8.5 h	minor adverse effects
Aceneuramic Acid	ZINC000004214715	131-48-6	DB11797	NCT01517880 NCT02377921	6 g triple daily	Orally Extended-Release	-	No serious adverse events

				NCT02845609				
(*S*)-Boceprevir	ZINC000014210455	394730-60-0	DB08873<br>DB05665	NCT01663922<br>NCT00910624<br>NCT02113631<br>NCT01912495<br>NCT01482767	400-800 mg single dose	Orally	3.4 h	fatigue, anemia, nausea, headache, and dysgeusia
(*R*)-Boceprevir	ZINC000014210457	394730-60-0	DB08873<br>DB05665	NCT01663922<br>NCT01945294		Orally		fatigue, anemia, nausea, headache, and dysgeusia
